# Critical illness-related corticosteroid insufficiency during difficult weaning from mechanical ventilation

**DOI:** 10.1186/s13613-021-00852-2

**Published:** 2021-04-26

**Authors:** François Bagate, Alexandre Bedet, Françoise Tomberli, Florence Boissier, Keyvan Razazi, Nicolas de Prost, Guillaume Carteaux, Armand Mekontso Dessap

**Affiliations:** 1 AP-HP, DHU A-TVB, Service de Médecine Intensive Réanimation, Centre Hospitalo-Universitaire Henri Mondor, 51, avenue du Mal de Lattre de Tassigny, 94010 Créteil Cedex, France; 2 Groupe de Recherche Clinique CARMAS, Faculté de Médecine, Université Paris Est Créteil, 94010 Créteil, France; 3 INSERM U955, Institut Mondor de Recherche Biomédicale, 94010 Créteil, France; 4 Service de Réanimation Médicale, CHU de Poitiers, Poitiers, France; 5 INSERM CIC 1402 (ALIVE Group), Université de Poitiers, Poitiers, France

**Keywords:** CIRCI, Mechanical ventilation, Difficult weaning, WiPO

## Abstract

**Background:**

Critical illness-related corticosteroid insufficiency (CIRCI) is common during critical illness and is usually associated with poor outcomes, as prolonged duration of mechanical ventilation (MV) and higher mortality. CIRCI may alter cardiac and vascular functions. Weaning-induced pulmonary oedema (WiPO) is a major mechanism of weaning failure. The aim of this study was to evaluate the role of CIRCI in patients with difficult ventilator weaning and its possible relation with WiPO.

**Methods:**

This is a prospective study conducted in the intensive care of a university hospital in France. Patients under MV for more than 24 h, meeting weaning criteria and having failed the first spontaneous breathing trial (SBT) underwent a corticotropin stimulation test, with assessment of total blood cortisol levels immediately before (*T*_0_) 0.25 mg iv of tetracosactrin and 30 and 60 min afterward. Δ_max_ was defined as the difference between the maximal value after the test and *T*_0_. CIRCI was defined as *T*_0_ < 10 μg/dL (276 nmol/L) and/or Δ_max_ < 9 μg/dL (248 nmol/L) and inadequate adrenal reserve as Δ_max_ < 9 μg/dL. Biomarkers (natriuretic peptide and protidemia) sampling and echocardiograms were performed during the second SBT and were used to diagnose WiPO, which was defined according to two definitions (one liberal and one conservative) derived from recent publications on the topic. Successful extubation was defined as patient alive without reintubation 7 days after extubation. A competing risk analysis was used to assess extubation failure and mortality.

**Results:**

Seventy-six consecutive patients (63 ± 14 years; 49 men) with difficult weaning were enrolled. CIRCI and inadequate adrenal reserve occurred in 25 (33%) and 17 (22%) patients, respectively. The probability of successful extubation was significantly decreased in patients with CIRCI or inadequate adrenal reserve, as compared to their counterparts, and this association persisted after adjustment on severity (SOFA score at first SBT). WiPO occurred in 44 (58%) and 8 (11%) patients, according to the liberal and conservative definition, respectively. WiPO was not associated with CIRCI nor with inadequate adrenal reserve.

**Conclusion:**

CIRCI was common during difficult weaning and was associated with its prolongation. We did not find a significant association between CIRCI and WiPO.

**Supplementary Information:**

The online version contains supplementary material available at 10.1186/s13613-021-00852-2.

## Introduction

Critical illness-related corticosteroid insufficiency (CIRCI) was described in intensive care unit (ICU) patients [1], with an impairment of the hypothalamo–pituitary–adrenal (HPA) axis without anatomical lesions. Latest guidelines were unable to reach agreement on a single test that can reliably diagnose CIRCI [2], although delta cortisol (change in baseline cortisol at 60 min of < 9 μg/dL) after the corticotropin stimulation test [250-μg Adreno CorticoTropic Hormone (ACTH) stimulation test] and a random plasma cortisol of < 10 μg/dL may be used by clinicians. Although the impairment of the HPA axis in critically ill patient is difficult to explore, the stimulation test seems to be useful in establishing a prognostic classification.

Some adrenal profiles derived from corticotropin stimulation test are usually associated with poor outcomes, as alteration of cardiac and vascular functions, prolonged duration of mechanical ventilation (MV) and higher mortality [3, 4]. The pathophysiology of weaning failure is complex [5], but weaning-induced pulmonary oedema (WiPO) is one of its major established causes [6, 7]. During the weaning process, the use of spontaneous breathing trial (SBT) is a real stress test [8], which may imply elevation of cortisol level to meet the cardiorespiratory physiological demand. The HPA axis may, therefore, play a major role in the response to the stress induced by ventilator weaning. Because adrenal insufficiency may impair cardiac function [9], we hypothesized that CIRCI may increase WiPO incidence. Conversely, an inadequate adrenal response may be associated with difficult weaning. Studies on the role of adrenal function in weaning failure are scarce [10]. The aims of this study were to evaluate the prevalence of adrenal insufficiency during difficult weaning from MV, and its possible relation with WiPO.

## Methods

### Study population

This ancillary study, planned a priori, was performed in one (Henri Mondor University hospital, Creteil, France) of the four centers participating in a prospective multicenter study assessment of WiPO and weaning-induced myocardial ischemia (WiCI) [11]. Patients screened for enrolment were those intubated for at least 24 h with ventilator settings allowing to initiate the weaning process [SpO_2_ > 90% or PaO_2_/FiO_2_ ≥ 150 mmHg with a fraction of inspired oxygen (FiO_2_) ≤ 40% and a positive end-expiratory pressure (PEEP) ≤ 8 cmH_2_O]. Exclusion criteria included age < 18 years, decision to withdraw life support, hemodynamic instability with significant doses of vasopressors (dopamine or dobutamine > 10 µg/kg/min, epinephrine or norepinephrine > 0.5 mg/h), patient deeply comatose or sedated, extreme temperatures (< 36 °C or > 39 °C) and patients with long-term corticosteroid therapy or under corticosteroid supplementation at time of corticotropin stimulation test. Criteria for SBT failure were respiratory rate ≥ 35 breaths/minute or increase ≥ 50%, SpO_2_ ≤ 90% or PaO_2_ ≤ 50 mmHg (with FiO_2_ ≥ 50%), heart rate ≥ 140 beats/minute, new-onset of supraventricular or ventricular arrhythmia, systolic arterial pressure > 180 or < 90 mmHg, alteration of consciousness, diaphoresis or any signs of respiratory distress [12, 13]. Patients who failed the first SBT were included in the study. A second SBT, consisting of a 2-h T-piece trial [14] was performed within 24 h after the first SBT in all included patients. Criteria for second SBT failure were the same as for the first SBT. Patients who succeeded the second SBT were extubated.

This study was conducted in accordance with the amended Declaration of Helsinki. The protocol was approved by our institution’s local ethics committee (Comité de Protection des Personnes Ile-de-France IX, approval number 10-064). The protocol was considered a component of standard care and the patient’s consent was waived. Written and oral information about the study were given to patients or families.

### Cortisol assays

As suggested by the guidelines [2], we used the 250-μg ACTH stimulation rather than the 1-μg ACTH stimulation test for the diagnosis of adrenal insufficiency for the following reasons. First, the accuracy of the low- and high-dose ACTH tests seems to be comparable [15]. Second, practical modalities of the 1-μg ACTH stimulation test are very complex.

The short corticotropin stimulation test was performed on the morning following the first SBT, by injecting 250 mg of tetracosactrin (Synacthen, Ciba, Reuil-Malmaison, France) intravenously as described previously [16]. The maximal post-stimulation concentration (*T*_max_) was the highest value between *T*_30_ and *T*_60_. The maximal cortisol response (Δ_max_) was defined as the difference between *T*_max_ and *T*_0_. CIRCI was defined as *T*_0_ < 10 μg/dL (276 nmol/L) and/or Δ_max_ < 9 μg/dL (248 nmol/L) [2]. We defined inadequate adrenal reserve as Δ_max_ < 9 μg/dL, whatever the baseline cortisol [3, 17]. Additional thresholds for adrenal insufficiency [*T*_0_ < 25 μg/dL (694 nmol/L) and Δ_max_ < 9 μg/dL (248 nmol/L)] were also tested, as proposed by Huang and Lin in a study focusing on ventilator weaning [10].

### Weaning-induced pulmonary oedema

To characterize cardiac function, transthoracic echocardiography was performed just before and at the end of the second SBT, as previously described [18]. Several biomarkers (B-type natriuretic peptide, troponin, protein) were measured during second SBT to assess WiPO. Because there is no non-invasive consensual definition of WiPO, we considered three criteria proposed in the recent literature: (i) echocardiographic signs of increased left atrial pressure at the end of the SBT: *E*/*A* ratio > 0.95 and *E*/*e*′ ratio > 8.5 [19]; (ii) an increase of BNP (absolute change ≥ 48 ng/L) or NT-proBNP (absolute change ≥ 21 ng/L) concentration during the SBT [20]; and (iii) an increase of protein concentration (relative change > 6%) during the SBT [21]. We further combined these criteria into two definitions of WiPO, as follows: a conservative definition (when at least two criteria were fulfilled), and a liberal definition (when at least one criterion was fulfilled) [11]. As per our weaning protocol and current guidelines [13], we performed a cuff leak test in selected patients at high risk of post-extubation stridor; no patient required a preventive or curative steroid treatment for stridor.

### Classification of weaning

Successful weaning was defined as patient alive and not reintubated within the 7 days following extubation, irrespective of the use of non-invasive ventilation [13]. We classified patients into three groups, according to the WIND classification [22], as follows: short weaning (successful weaning or death within 1 day after the first SBT), difficult weaning (successful weaning or death after more than 1 day but in less than 7 days after the first SBT), and prolonged weaning (successful weaning or death after 7 days following the first SBT).

### Statistical analysis

The data were analyzed using the JMP software (version 9; SAS Institute Inc, Cary, NC). Categorical variables were expressed as numbers (percentage) and continuous data as medians (25th–75th percentiles). We used the Chi-squared or Fisher exact test to compare categorical variables between groups and the Student’s *T* test, Mann–Whitney test or Wilcoxon paired test to compare continuous variables, as appropriate. We also used the Kaplan–Meier method to assess the effect of adrenal function on the cumulative probability of successful extubation. Since weaning outcome and death act as competing risks, we designed the present work with a competing risk approach to properly estimate the effect of adrenal function on extubation outcome.

### Competing risks analysis

A competing risk is an event whose occurrence either precludes the occurrence of another event under examination or fundamentally alters the probability of occurrence of this other event [23]. Death is a competing risk for weaning outcome. Indeed, patients are no longer at risk for weaning failure after death. In this context, standard survival methods (Kaplan–Meier method and Cox model) are inappropriate, because they assume that censoring is non-informative and specific competing risk methods need to be considered. We, therefore, used a competing risk model (cumulative incidence function of the Gray model) [24] to properly estimate the effect of adrenal function on weaning outcome (up to day 28), while considering death as a competing event. The strength of the association between each variable and the outcome was assessed using the sub-hazard ratio associated with the cumulative incidence function estimated using the cmprsk package developed by Gray in the R software (http://biowww.dfci.harvard.edu/~gray/cmprsk_2.1-4.tar.gz). Two-sided *p* values < 0.05 were considered significant.

## Results

### Patient population and ventilator weaning

**Fig. 1:**
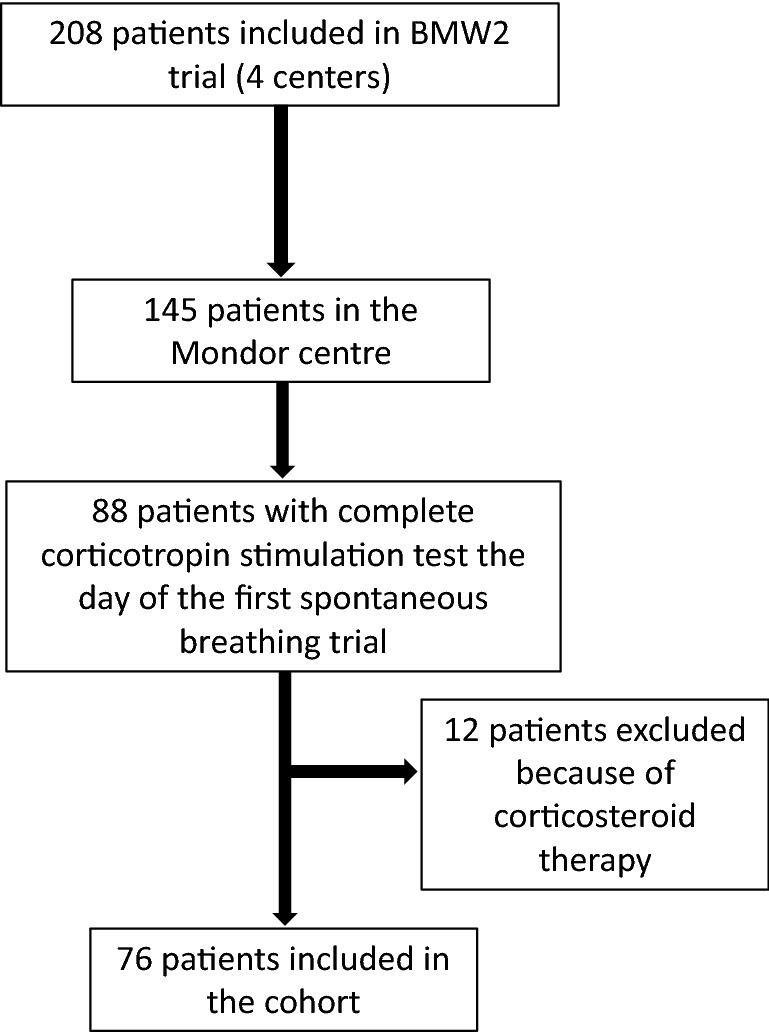
Flow-chart

Among the 145 participants enrolled, a short corticotropin stimulation test could not be completed in 57 patients. Twelve patients were further excluded because of corticosteroid therapy (Fig. 1). Among the 76 patients included in this study, 50 failed (66%) the second SBT while 26 succeeded (34%). Among included patients, 19 (25%) failed extubation. According to the WIND classification [22], weaning was short in 21 patients (28%), difficult in 33 patients (43%) and prolonged in 22 patients (29%). Patient characteristics were similar between simple, difficult and prolonged weaning except for a higher prevalence of heart failure with reduced ejection fraction, septic shock, and ventilator-associated pneumonia before weaning and a longer duration of MV and fluid balance before weaning in patients with prolonged weaning as compared to their counterparts (Table 1). Patients with prolonged weaning exhibited a higher total duration of MV, ICU length of stay, hospital length of stay, and ICU mortality as compared to their counterparts (Table 1).

**Table 1:** Clinical and biological characteristics of patients according to WIND classification

Variables	Total (*n* = 76)	WIND classification			*p* value
		Simple (*n* = 21)	Difficult (*n* = 33)	Prolonged (*n* = 22)	
Age (years)	76	61.0 (± 14.2)	63.4 (± 13.2)	63.2 (± 14.3)	0.81
Female gender	76	6 (28.6%)	13 (39.4%)	8 (36.4%)	0.72
Body mass index (kg/m^2^)	61	26.0 (23.5–30.0)	28.6 (22.8–35.7)	25.6 (22.9–32.5)	0.64
SAPS-II at admission	76	54 (34–65)	53 (41–71)	50 (41–70)	0.43
Comorbidities					
COPD	76	3 (14.7%)	10 (30.3%)	7 (31.8%)	0.34
Restrictive lung disease	76	2 (9.5%)	6 (18.2%)	1 (4.6%)	0.29
Obstructive sleep apnea syndrome	76	6 (28.6%)	7 (21.2%)	1 (4.6%)	0.11
Asthma	76	2 (9.5%)	0 (0%)	1 (4.6%)	0.21
Current smoker	76	9 (42.9%)	16 (48.5%)	15 (68.2%)	0.21
Central nervous system disease	76	2 (9.5%)	4 (12.1%)	3 (13.6%)	0.91
Peripheral neuropathy	76	0 (0%)	1 (3.0%)	1 (4.6%)	0.64
Mental illness	76	2 (9.5%)	3 (9.1%)	1 (4.6%)	0.79
HF with preserved ejection fraction	76	6 (28.6%)	8 (24.2%)	3 (13.6%)	0.47
HF with reduced ejection fraction	76	1 (4.8%)	3 (9.1%)	7 (31.8%)	0.02
Atrial fibrillation	76	2 (9.5%)	8 (24.2%)	7 (31.8%)	0.20
Arterial hypertension	76	12 (57.1%)	20 (60.6%)	11 (50%)	0.74
Valvular heart disease	76	4 (19.1%)	4 (12.1%)	3 (13.6%)	0.77
Coronary artery disease	76	5 (23.8%)	6 (18.2%)	6 (27.3%)	0.72
Pulmonary hypertension	76	1 (4.8%)	3 (9.1%)	1 (4.6%)	0.74
Reason for mechanical ventilation	76				0.46
Coma		3 (14.7%)	4 (12.1%)	1 (4.6%)	
Septic shock		3 (14.7%)	2 (6.1%)	6 (27.3%)	
COPD exacerbation		2 (9.5%)	2 (6.1%)	1 (4.6%)	
Pneumonia		3 (14.7%)	10 (30.3%)	5 (22.7%)	
Cardiogenic pulmonary oedema		3 (14.7%)	2 (6.1%)	1 (4.6%)	
Cardiac arrest		3 (14.7%)	8 (24.2%)	2 (9.1%)	
Surgery		1 (4.8%)	4 (12.1%)	3 (13.6%)	
Others		3 (14.7%)	1 (3.0%)	3 (13.6%)	
Complications in ICU before weaning					
Acute respiratory distress syndrome	76	7 (33.3%)	12 (36.4%)	12 (54.6%)	0.29
Septic shock	76	9 (42.9%)	12 (36.4%)	16 (72.7%)	0.03
Ventilator-associated pneumonia	76	4 (19.1%)	9 (27.3%)	13 (59.1%)	0.01
Atrial fibrillation	76	4 (19.1%)	9 (27.3%)	11 (50%)	0.11
Ventilation duration before first SBT (d)	76	4 (3–10)	4 (2–11)	13 (4–20)	< 0.01
Fluid balance before first SBT (L)	76	3.7 (0.9–9.3)	3.5 (0.9–9.7)	9.2 (2.4–18.5)	0.046
SOFA before weaning	76	3 (2–7)	3 (2–5)	4 (3–6)	0.19
Success of second SBT	76	12 (57.1%)	11 (33.3%)	3 (13.6%)	0.01
Biological data before weaning					
Protidemia (g/L)	76	60.7 (± 10.0)	60.2 (± 10.0)	60.8 (± 8.0)	0.97
Creatinine (μmol/L)	76	87 (75–128)	85 (58–157)	75 (56–123)	0.34
Hemoglobin (g/dL)	76	10.0 (8.1–11.0)	9.5 (8.3–10.9)	8.6 (7.8–10.6)	0.27
White blood count (g/L)	76	12.8 (9.1–17.6)	10.1 (8.6–14.5)	12.7 (8.4–18.2)	0.49
Procalcitonin (mg/L)	73	1.1 (0.5–5.0)	0.5 (0.2–1.4)	0.6 (0.2–1.5)	0.15
Outcomes					
Success extubation	76	21 (100%)	30 (90.9%)	6 (27.3%)	< 0.01
Weaning duration (d)	76	1 (1–1)	3 (2–5)	17 (9–33)	< 0.01
Ventilation duration (d)	76	5 (3–7)	7 (6–14)	33 (20–53)	< 0.01
ICU length of stay (d)	76	7 (5–12)	13 (9–20)	35 (26–58)	< 0.01
Hospital length of stay (d)	76	20 (13–30)	23 (15–49)	59 (32–)	< 0.01
ICU mortality	76	0 (0%)	3 (9.1%)	11 (50%)	< 0.01

Values are expressed as mean (± SD) or median (IQR) as appropriate

*SAPS-II* Simplified Acute Physiology Score II, *COPD* chronic obstructive pulmonary disease, *HF* heart failure, *SBT* spontaneous breathing trial, *SOFA score* Sequential Organ Failure Assessment, *ICU* intensive care unit

### Adrenal function and ventilator weaning

Patient characteristics were similar between patients with CIRCI and those without, except for more atrial fibrillation and less restrictive lung disease and obstructive sleep apnea syndrome in the former group (Additional file 1: Table S1). Regarding the outcomes, patients with CIRCI had a higher rate of extubation failure and mortality (Additional file 1: Table S1).

### Association between adrenal function and ventilator weaning

*T*_0_ and *T*_max_ were similar between weaning groups, whereas Δ_max_ was lower in patients with prolonged weaning as compared to other groups (Table 2). CIRCI, inadequate adrenal reserve, and adrenal insufficiency (as per Hang and Lin definition) occurred in 25 (33%), 17 (22%), and 10 (13%) patients, respectively (Table 2). Inadequate adrenal reserve and adrenal insufficiency (as per Hang and Lin definition) were more frequent in the prolonged weaning group as compared to other weaning groups, whereas the difference did not reach statistical significance for CIRCI (Table 2). We found no association between adrenal function and WiPO, whatever the definition used for adrenal function and for WiPO (Table 3).

**Table 2:** Adrenal function of patients according to WIND classification

Variables	Total (*n* = 76)	WIND classification			*p* value
		Simple (*n* = 21)	Difficult (*n* = 33)	Prolonged (*n* = 22)	
*T*_0_ (nmol/L)	655 (438–814)	623 (373–833)	690 (391–849)	615 (482–810)	0.84
*T*_max_ (nmol/L)	977 (794–1356)	1055 (896–1317)	1039 (763–1431)	904 (753–1204)	0.36
Δ_max_ (nmol/L)	429 (267–574)	556 (429–678)	408 (287–638)	261 (151–484)	< 0.01
CIRCI	25 (32.9%)	5 (23.8%)	9 (27.3%)	11 (50%)	0.12
Inadequate adrenal reserve	17 (22.4%)	2 (9.5%)	4 (12.1%)	11 (50%)	< 0.01
AI (Huang and Lin definition [8])	10 (13.2%)	0	1 (3.0%)	9 (40.9%)	< 0.01

Values are expressed as mean (± SD) or median (IQR) as appropriate

*T*_0_: baseline plasma cortisol (before short corticotropin test); *T*_max_: maximal cortisol post-stimulation; Δ_max_: maximal cortisol response; CIRCI: critical illness-related corticosteroid insufficiency = *T*_0_ < 10 μg/dL (276 nmol/L) and/or Δ_max_ < 9 μg/dL (248 nmol/L); inadequate adrenal reserve: Δ_max_ < 9 μg/dL (248 nmol/L); AI: adrenal insufficiency (Huang and Lin definition) = *T*_0_ < 25 μg/dL (694 nmol/L) and Δ_max_ < 9 μg/dL (248 nmol/L)

**Table 3:** Weaning-induced pulmonary oedema according to adrenal reserve

Variables	WiPO classification					
	Conservative definition		*p* value	Liberal definition		*p* value
	Yes (*n* = 8)	No (*n* = 68)		Yes (*n* = 44)	No (*n* = 32)	
*T*_0_ (nmol/L)	833 (480–894)	637 (438–800)	0.18	675 (458–875)	611 (322–791)	0.17
*T*_0_ < 276 nmol/L	0 (0%)	9 (13.2%)	0.58	3 (6.8%)	6 (18.8%)	0.15
*T*_max_ (nmol/L)	1136 (896–1439)	977 (781–1325)	0.47	1020 (896–1414)	893 (726–1322)	0.08
Δ_max_ (nmol/L)	432 (201–519)	429 (267–614)	0.51	466 (279–610)	404 (222–574)	0.49
Δ_max_ < 248 nmol/L	2 (25%)	15 (22.1%)	> 0.99	9 (20.5%)	8 (25%)	0.64
AI (Huang and Lin def)	0 (0%)	10 (14.7%)	0.59	5 (11.4%)	5 (15.6%)	0.59
CIRCI	2 (25%)	23 (33.8%)	> 0.99	12 (27.3%)	13 (40.6%)	0.22

Values are expressed as mean (± SD) or median (IQR) as appropriate

WiPO: weaning-induced pulmonary oedema, *T*_0_: baseline plasma cortisol (before short corticotropin test), *T*_max_: maximal cortisol post-stimulation, Δ_max_: maximal cortisol response, AI: adrenal insufficiency (Huang and Lin definition) = *T*_0_ < 25 μg/dL (694 nmol/L) and Δ_max_ < 9 μg/dL (248 nmol/L), CIRCI: critical illness-related corticosteroid insufficiency = *T*_0_ < 10 μg/dL (276 nmol/L) and/or Δ_max_ < 9 μg/dL (248 nmol/L)

**Fig. 2:**
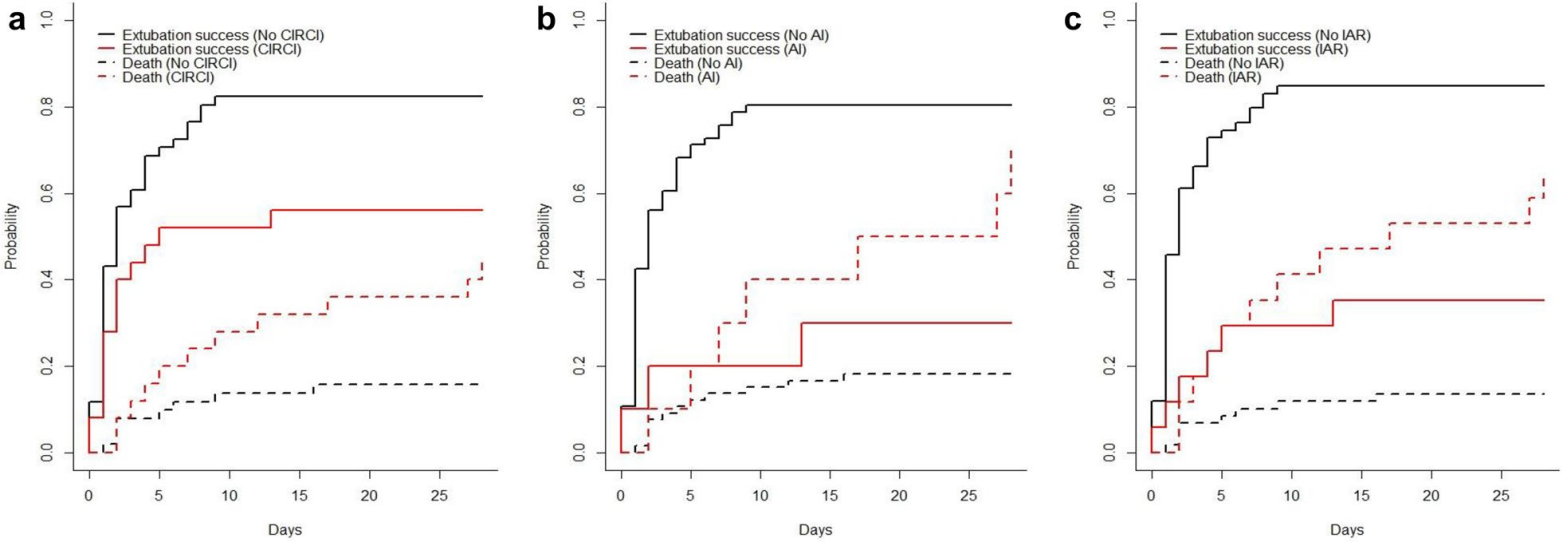
Cumulative probability of extubation success and death in patients with altered adrenal function (in red) and preserved adrenal function (in black). Time from weaning to extubation success (continuous line), and to death (dashed line) were handled as competing risks. Altered adrenal function was assessed as CIRCI (**a**), adrenal insufficiency (as per Hang and Lin definition) (**b**) or inadequate adrenal reserve (**c**)

From the Fine and Gray model, the probability of successful extubation during the 28 days following inclusion was significantly lower in patients with altered adrenal function while adjusting for death as a competing event: sub-hazard ratios of 0.54 (0.30–0.97), *p* = 0.04, 0.24 (0.08–0.75), *p* = 0.02, and 0.25 (0.11–0.57), *p* < 0.01, for CIRCI (Fig. 2a), adrenal insufficiency (as per Hang and Lin definition) (Fig. 2b), and inadequate adrenal reserve (Fig. 2c), respectively. Conversely, the probability of death was significantly higher in patients with altered adrenal function while adjusting for weaning outcome as a competing event: sub-hazard ratios of 3.10 (1.26–7.64), *p* = 0.01, 4.42 (1.95–10.0), *p* < 0.001, and 6.04 (2.46–14.8), *p* < 0.0001 for CIRCI (Fig. 2a), adrenal insufficiency (as per Hang and Lin definition) (Fig. 2b), and inadequate adrenal reserve (Fig. 2c), respectively.

## Discussion

We herein report in this cohort of patients who failed their first SBT: (i) a high prevalence of altered adrenal function; and (ii) an association between altered adrenal function and extubation failure, but not with WiPO.

### Adrenal insufficiency and corticosteroid therapy during ventilator weaning

Several studies reported on adrenal insufficiency in patients under prolonged MV, present in about 30–75% of cases, according to the definitions used [4, 10]. This adrenal insufficiency was associated with a longer weaning period [4, 10] and a higher mortality [25]. Moreover, in recent randomized controlled trials assessing the benefit of corticosteroid therapy in septic shock, time to weaning from MV was shorter in the intervention group than the placebo group [26, 27]. However, these studies and ours do not allow to use CIRCI to select for glucocorticoid replacement during ventilator weaning. The usefulness of corticosteroid on weaning from mechanical ventilation could be mediated by hemodynamic improvement and/or inflammation blunting. To date, only one study had specifically evaluated adrenal insufficiency during ventilator weaning [10]. In this cohort of patients under MV for more than 72 h, patients were assessed for adrenal function and those with adrenal insufficiency were randomized to receive 50 mg intravenous hydrocortisone every 6 h or a placebo. In this trial [10], extubation success was comparable in the adequate adrenal reserve group (88%) and intervention group (91%); and these rates were both higher than those of the placebo group (69%), the latter having a longer weaning duration. The pathophysiological mechanisms underlying these results remain unclear.

### Physiologic implications of CIRCI during weaning

Based on the known circulatory effect of steroids in septic shock patients [28], the main hypothesis proposed [10], is that stress dose of hydrocortisone may reduce weaning failure by improving hemodynamic stability. Our research group previously showed a close association between adrenal insufficiency and cardiac failure in patients with cardiogenic shock or septic cardiomyopathy [16, 29]. However, we found no association between CIRCI and WiPO in the present cohort. CIRCI may affect weaning outcome via other pathways of stress response. Weaning-induced stress response has been highlighted in different critical care settings including neonatal [30], surgical [31], or medical [6] ICU patient groups. Besides the cardiovascular system, the stress response may involve other major functions of the respiratory or immunologic system. Steroid stress dose administration may improve altered immune response in patients undergoing weaning [28, 32, 33], but we did not specifically assess ventilator-associated pneumonia during the weaning phase.

### Strengths and limitations

Strengths of our study include its prospective design and carefully assessment of WiPO using echocardiography and biomarkers. Limitations of our study include the monocentric setting and the limited sample size, which precluded any robust multivariable analysis of factors associated with extubation failure or CIRCI. In addition, several potential confounding factors can limit the interpretation of these results. Etomidate is a common hypnotic agent, frequently used in rapid sequence intubation in our center and the best-known drug to interfere with cortisol metabolism. A single bolus of etomidate is a major determinant of CIRCI for at least 24–48 h [25, 34]. However, the possible influence of this drug on our data is minimized by the systematic use of etomidate in all intubated patients, and the relatively long delay (median of 5 days) between intubation and the initiation of weaning. Moreover, some patients (*n* = 10, 13%) transiently receiving steroids (not long-term users and not currently under steroids at time of stimulation test) were included and may constitute a limit. However, their adrenal profile (*T*_0_ and Δ_max_) did not significantly differ from that of their counterparts. Finally, the more important limitation is probably the interpretation of the corticotropin stimulation test in these critically ill patients during a de-escalation phase. Measure of total plasmatic cortisol may not accurately reflect his real activity, especially in hypoproteinemic patients. Although we found no difference in protidemia level according to adrenal function, free cortisol is probably more suitable [35]. Furthermore, Peeters et al. [36] have recently suggested that corticotropin stimulation test may not provide reliable information on adrenal insufficiency and may not exclude CIRCI in critically ill patients after a prolonged ICU stay (more than 7 days). In the specific population of critically ill patients with difficult weaning, further studies are warranted with plasmatic free cortisol to confirm our results.

## Conclusion

In conclusion, alteration of the HPA axis was common during difficult weaning and associated with poor weaning outcome. We found no association between CIRCI and WiPO. Further studies are needed to scrutinize the role of adrenal function during weaning.

## Supplementary Information

**Additional file 1: Table S1.** Clinical and biological characteristics of patients according to CIRCI.

## Data Availability

All data generated and analyzed during the study are included in the published article and can be shared upon request. All authors helped to revise the draft of the manuscript.

## Abbreviations

CIRCI: Critical illness-related corticosteroid insufficiency
ICU: Intensive care units
HPA: Hypothalamo–pituitary–adrenal
MV: Mechanical ventilation
WiPO: Weaning-induced pulmonary oedema
SBT: Spontaneous breathing trial
WiCI: Weaning-induced myocardial ischemia
FiO_2_: Fraction of inspired oxygen
PEEP: Positive end-expiratory pressure
*T*_max_: Maximal post-stimulation concentration
Δ_max_: Maximal cortisol response
*T*_0_: Baseline cortisol concentration
BNP: B-type natriuretic peptide
