# Supplementary material for: Critical illness-related corticosteroid insufficiency during difficult weaning from mechanical ventilation
Source: Ann Intensive Care. 2021 Apr 26;11:65. doi: 10.1186/s13613-021-00852-2 (PMC8072727; doi:10.1186/s13613-021-00852-2)
Supplement: Supplementary file 1 — Additional file 1: Table S1. Clinical and biological characteristics of patients according to CIRCI. [file 13613_2021_852_MOESM1_ESM.docx]

| **Table S1 Clinical and biological characteristics of patients according to CIRCI** | | | | | |
| --- | --- | --- | --- | --- | --- |
| **Variables** | **n** | **Total**  **(n=76)** | **CIRCI**  **(n=25)** | **No CIRCI**  **(n=51)** | ***p* value** |
| Age (years) | 76 | 62.7 (+/-13.6) | 66.8 (+/-12.2) | 60.6 (+/-14.0) | 0.053 |
| Female gender | 76 | 27 (35.5%) | 7 (28.0%) | 20 (39.2%) | 0.34 |
| Body mass index (Kg/m^2^) | 61 | 26.7 (23.0-32.5) | 27.5 (23.4-33.3) | 26.3 (22.7-32.6) | 0.89 |
| SAPS-II at admission | 76 | 53 (39-69) | 50 (38-65) | 53 (42-69) | 0.35 |
| ***Comorbidities*** |  |  |  |  |  |
| COPD | 76 | 20 (26.3%) | 6 (24.0%) | 14 (27.5%) | >0.99 |
| Restrictive lung disease | 76 | 9 (11.8%) | 0 (0%) | 9 (17.7%) | 0.03 |
| Obstructive sleep apnea syndrome | 76 | 14 (18.4%) | 1 (4.0%) | 13 (25.5%) | 0.03 |
| Asthma | 76 | 3 (3.9%) | 2 (8.0%) | 1 (2.0%) | 0.25 |
| Current smoker | 76 | 40 (52.6%) | 14 (56.0%) | 26 (51.0%) | 0.68 |
| Central nervous system disease | 76 | 9 (11.8%) | 2 (8.0%) | 7 (13.7%) | 0.71 |
| Peripheral neuropathy | 76 | 2 (2.6%) | 1 (4.0%) | 1 (2.0%) | >0.99 |
| Mental illness | 76 | 6 (7.9%) | 2 (8.0%) | 4 (7.8%) | >0.99 |
| HF with preserved ejection fraction | 76 | 17 (22.4%) | 7 (28.0%) | 10 (19.6%) | 0.41 |
| HF with reduced ejection fraction | 76 | 11 (14.5%) | 5 (20.0%) | 6 (11.8%) | 0.34 |
| Atrial fibrillation | 76 | 17 (22.4%) | 9 (36.0%) | 8 (15.7%) | 0.046 |
| Arterial hypertension | 76 | 43 (56.6%) | 14 (56.0%) | 29 (56.9%) | 0.94 |
| Valvular heart disease | 76 | 11 (14.5%) | 4 (16.0%) | 7 (13.7%) | >0.99 |
| Coronary artery disease | 76 | 17 (22.4%) | 8 (32.0%) | 9 (17.7%) | 0.16 |
| Pulmonary hypertension | 76 | 5 (6.6%) | 1 (4.0%) | 4 (7.8%) | >0.99 |
| ***Reason for mechanical ventilation*** | 76 |  |  |  | 0.25 |
| Coma |  | 8 (10.5%) | 5 (20.0%) | 3 (5.9%) |  |
| Septic shock |  | 11 (14.5%) | 2 (8.0%) | 9 (17.7%) |  |
| COPD exacerbation |  | 5 (6.6%) | 2 (8.0%) | 3 (5.9%) |  |
| Pneumonia |  | 18 (23.7%) | 8 (32.0%) | 10 (19.6%) |  |
| Cardiogenic pulmonary oedema |  | 6 (7.9%) | 2 (8.0%) | 4 (7.8%) |  |
| Cardiac arrest |  | 13 (17.1%) | 2 (8.0%) | 11 (21.6%) |  |
| Surgery |  | 8 (10.5%) | 1 (4.0%) | 7 (13.7%) |  |
| Others |  | 7 (9.2%) | 3 (12.0%) | 4 (7.8%) |  |
| ***Complications in ICU before weaning*** |  |  |  |  |  |
| Acute respiratory distress syndrome | 76 | 31 (40.8%) | 9 (36.0%) | 22 (43.1%) | 0.55 |
| Septic shock | 76 | 37 (48.7%) | 13 (52.0%) | 24 (47.1%) | 0.69 |
| Ventilator-associated pneumonia | 76 | 26 (34.2%) | 8 (32.0%) | 18 (35.3%) | 0.78 |
| Atrial fibrillation | 76 | 24 (31.6%) | 10 (40.0%) | 14 (27.5%) | 0.27 |
| Ventilation duration before first SBT (d) | 76 | 5 (2-13) | 4 (3-13) | 5 (2-13) | 0.66 |
| Fluid balance before first SBT (L) | 75 | 4.4 (1.7-10.4) | 6.1 (0.9-15.7) | 4.3 (1.9-9.7) | 0.51 |
| SOFA before weaning | 76 | 3 (3-5) | 4 (3-8) | 3 (2-5) | 0.053 |
| Success of second SBT | 76 | 26 (34.2%) | 8 (32.0%) | 18 (35.3%) | 0.78 |
| ***Biological data before weaning*** |  |  |  |  |  |
| Protidemia (g/L) | 76 | 60.5 (+/-9.3) | 60.5 (+/-9.0) | 60.5 (+/-9.6) | 0.99 |
| Creatinine (μmol/L) | 76 | 82 (63-140) | 89 (63-156) | 82 (62-132) | 0.76 |
| Hemoglobin (g/dL) | 76 | 9.4 (7.9-10.8) | 9.1 (7.7-11.5) | 9.5 (8.2-10.6) | 0.63 |
| White blood count (G/L) | 76 | 12.2 (8.7-16.8) | 10.5 (7.7-13.9) | 12.8 (8.9-17.8) | 0.09 |
| Procalcitonin (mg/L) | 73 | 0.6 (0.3-1.7) | 0.7 (0.3-2.9) | 0.6 (0.2-1.4) | 0.58 |
| ***Outcomes*** |  |  |  |  |  |
| Success extubation | 76 | 57 (75.0%) | 14 (56.0%) | 43 (84.3%) | <0.01 |
| Weaning duration (d) | 76 | 3 (1-8) | 4 (2-17) | 2 (1-7) | 0.07 |
| Ventilation duration (d) | 76 | 11 (5-20) | 11 (6-32) | 11 (5-19) | 0.55 |
| ICU length of stay (d) | 76 | 14 (8-28) | 17 (10-32) | 14 (7-14) | 0.39 |
| Hospital length of stay (d) | 76 | 26 (17-59) | 29 (19-59) | 26 (15-59) | 0.65 |
| Mortality | 76 | 14 (18.4%) | 9 (36.0%) | 5 (9.8%) | <0.01 |
| Values are expressed as mean (±SD) or median (IQR) as appropriate. CIRCI: Critical Illness-Related Corticosteroid Insufficiency: T_0_<10μg/dL (276nmol/L) and/or Δmax<9μg/dL (248nmol/L), SAPS II: Simplified Acute Physiology Score II, COPD: chronic obstructive pulmonary disease, HF: heart failure, SBT: spontaneous breathing trial, SOFA score: Sequential Organ Failure Assessment, ICU: intensive care unit. | | | | | |
